# *Announcement:* Community Preventive Services Task Force Recommendations for Multicomponent Interventions to Increase Breast, Cervical, and Colorectal Cancer Screening

**DOI:** 10.15585/mmwr.mm6628a7

**Published:** 2017-07-21

The Community Preventive Services Task Force recently posted on its website new information regarding recommendations for multicomponent interventions for three different cancers: 1) “Increasing Cancer Screening: Multicomponent Interventions — Breast Cancer,” https://www.thecommunityguide.org/findings/cancer-screening-multicomponent-interventions-breast-cancer; 2) “Increasing Cancer Screening: Multicomponent Interventions — Cervical Cancer,” https://www.thecommunityguide.org/findings/cancer-screening-multicomponent-interventions-cervical-cancer; and 3) “Increasing Cancer Screening: Multicomponent Interventions — Colorectal Cancer,” https://www.thecommunityguide.org/findings/cancer-screening-multicomponent-interventions-colorectal-cancer.

Established in 1996 by the U.S. Department of Health and Human Services, the task force is an independent, nonfederal panel of public health and prevention experts who are appointed by the director of CDC. The task force provides information for a wide range of persons who make decisions about programs, services, and other interventions to improve population health. Although CDC provides administrative, scientific, and technical support for the task force, the recommendations developed are those of the task force and do not undergo review or approval by CDC.

